# Effects of extended and seasonal calving systems on milk composition and heat coagulation time in a pasture-based dairy herd

**DOI:** 10.3168/jdsc.2026-1003

**Published:** 2026-03-27

**Authors:** E. Bejarano, F. Harte, A. Garay, D. Ubios, A. Mendoza, A. Cartaya, S. Jorcin, T. López-Pedemonte

**Affiliations:** 1 Unidad Tecnológica de Lácteos, Universidad Tecnológica del Uruguay, 70200, La Paz, Colonia, Uruguay; 2 Department of Food Science, Pennsylvania State University, State College, PA 16802; 3 Instituto Nacional de Investigación Agropecuaria (INIA), Programa de Producción de Leche, Estación Experimental INIA La Estanzuela, 39173, Colonia, Uruguay

## Abstract

Animal management and environmental conditions influence milk composition and processing properties. Seasonal calving systems are used in milk production to optimize costs and align milk supply with forage availability; however, the effects of concentrating calvings in fall and winter, compared with extended calving, on milk composition and heat coagulation time (HCT) have not been clearly defined. The objective of this study was to evaluate whether calving strategy affects milk composition and HCT in a pasture-based dairy system. Thirty New Zealand Holstein cows were assigned to 3 calving systems—extended (EXT: March–October, spanning fall-winter with calvings distributed across both seasons), winter seasonal (WIN: June–August, concentrated in winter), and fall seasonal (FC: March–May, concentrated in fall)—with 10 cows per system, and monitored over one production year. Composite milk samples were collected in 4 seasonal windows (spring, summer, fall, and winter) and analyzed for composition (protein, casein, TS, SNF, urea, citric acid, lactose, and titratable acidity) and heat stability, assessed both as HCT at native pH and as HCT–pH profiles after adjustment to target pH values from 6.5 to 7.0. No single compositional variable consistently explained the observed HCT patterns across calving systems and seasons. In pooled linear regression models, HCT was positively associated with grazed pasture in the diet and days in milk, and negatively associated with DMI. These findings highlight the complexity of milk heat stability under pasture-based conditions and indicate that more detailed studies are needed to identify robust predictors of HCT.

## Highlights

• Fall or winter calving did not markedly change milk HCT in this herd.
• Forage availability and lactation stage were linked to milk composition.
• Milk HCT rose with pasture and days in milk, but declined with dry matter intake.
• Season and calving system did not alter the type-A HCT-pH profile.

## Graphical Abstract

**Figure:**
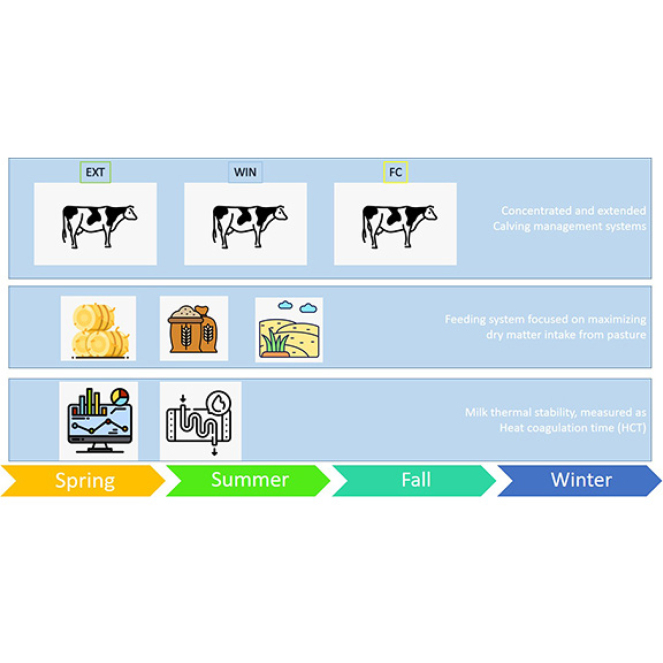
**Summary:**Milk compositional and processing quality traits are critical for the dairy industry, and management conditions such as calving pattern may influence these traits. We evaluated milk composition and heat stability (heat coagulation time [HCT]) over 4 seasonal sampling windows in 1 year in 3 pasture-based production systems: 2 with calvings concentrated in fall (FC) or winter (WIN) and 1 with extended calving (EXT). Calving system per se did not affect HCT. However, seasonal changes in grazed pasture inclusion and stage of lactation (days in milk) were directly positively associated with HCT, whereas higher dry matter intake tended to be linked with lower HCT. These findings suggest that, within the conditions of this study, differences in feeding and stage of lactation may be more closely associated with variation in milk heat stability than calving pattern per se. Icons from Flaticon (www.flaticon.com).

Milk composition and physicochemical properties are affected by a range of intrinsic and extrinsic factors, including breed, stage of lactation, parity, feeding practices, and cow health (Fox et al., 2015). In North America, year-round calving combined with TMR feeding generally results in herds with cows at different lactation stages throughout the year, contributing to a more stable milk supply and reducing the seasonality commonly observed in pasture-based systems (Schingoethe, 2017; Moscovici Joubran et al., 2021). In contrast, in Ireland and New Zealand, seasonal calving aligned with pasture growth leads to marked variations in milk composition throughout the lactation cycle (Timlin et al., 2021; ICBF, n.d.). In Uruguay and central-eastern Argentina, dairy farms are often classified as pasture-based, where ≥60% of DMI comes from pasture, or supplement-based, where <60% of DMI comes from pasture (MGAP-DIEA, 2021; Stirling et al., 2021; Pedemonte et al., 2024). In Uruguay, 16% of dairy systems manage year-round calving (≥5% of births per month), 60% adopt extended calving (March–October), and 24% use compact calving (>80% of births within <5 mo; Ubios and Mendoza, 2024). When considered by season, 45% of calvings occur in fall, 21% in winter, 26% in spring, and 8% in summer (MGAP-DIEA, 2021).

Milk technological properties are driven by changes in composition and physicochemical parameters, and heat stability, commonly assessed as heat coagulation time (**HCT**), is particularly important for the dairy industry because good performance on this trait is associated with milk powders and reconstituted products that do not coagulate during high-heat processing or shelf-life (Loveday et al., 2021). For example, Karlsson et al. (2019) reported that the extent of sedimentation in UHT milk during storage was inversely related to HCT measured in the corresponding raw milk. Research on the effects of concentrating calvings on technological properties of milk has been conducted in Ireland and New Zealand, where calvings are concentrated in January–April (Li et al., 2019) and late July–October (ICBF, n.d.), respectively. These windows coincide with the transition into spring in both countries, when favorable weather increases the supply of fresh forage (Moscovici Joubran et al., 2021). To our knowledge, no studies have specifically evaluated how fall- and winter-concentrated calving systems (**CS**), compared with an extended CS, affect milk composition and heat stability. Therefore, we compared these calving strategies—defined by the timing and concentration of calvings—to test their effects on milk composition (fat, protein, casein, lactose, TS, SNF, titratable acidity, citric acid, urea) and HCT.

The experiment was conducted at the dairy experimental station of the National Institute of Agricultural Research (INIA La Estanzuela, Colonia, Uruguay) over a single production year, with 4 seasonal sampling windows (**S**): **S1**, September 2023 (spring); **S2**, December 2023 (summer); **S3**, April 2024 (fall); and **S4**, July 2024 (winter). Animal care and handling procedures were carried out in accordance with the research protocols involving animals approved by the INIA Bioethics Committee of Animal Experimentation (protocol no. 2021.2). Within each season, milk was sampled over 3 consecutive weeks, on 3 sampling days per week. Three CS groups were evaluated using 10 New Zealand Holstein-Friesian cows per group: fall calving (**FC**, March–May), winter calving (**WIN**, June–August), and extended calving (**EXT**, March–August). Cows were randomly allocated to each CS (10 cows per CS) and balanced for parity, milk yield (L/d), and BW. For each CS, milk from the 10 cows was pooled at each milking, and morning and evening milkings were combined into a single daily composite sample of the group, which was used as the experimental unit for milk analyses. Composite samples were immediately cooled to 4°C and transported to the Dairy Science and Technology Unit at the Technological University of Uruguay (UTEC, Colonia La Paz, Uruguay) for analysis. Feeding management prioritized pasture intake, which was determined by weekly pasture growth rate, whereas the concentrate was offered individually in the milking parlor (1:3 of annual DMI) and the silage was offered as a buffer, in a feedpad, in case of pasture shortage (Stirling et al., 2021).

All milk samples were analyzed for physicochemical and technological properties. A 50-mL aliquot was used to determine TS, citric acid, and urea using a MilkoScan FT3 (Foss, Hiller⊘d, Denmark), following manufacturer guidelines. The instrument was calibrated semi-annually using Actalia-Cecalait reference material preserved with Bronopol (0.02%). Milk pH was measured at 22°C. The HCT was assessed at 140°C at native pH and pH-adjusted conditions (6.5 to 7.0), following the method of Davies and White (1966), with minor modifications. Three milliliters of skim milk was poured into borosilicate glass tubes (120 mm length × 10 mm external diameter × 3 mm wall thickness), sealed with silicone plugs, placed on a rocker, and immersed in an oil bath under continuous agitation (model 7722.0000, Hettich Benelux, Tuttlingen, Germany). The HCT was recorded as the time (min) to visible coagulation, with each sample measured in triplicate.

Data were screened for outliers (interquartile range; k = 1.5), normality (Shapiro–Wilk), and homoscedasticity (Levene). When assumptions were satisfied, we used one-way ANOVA to evaluate the effect of sampling window (season: S1–S4) within each CS. For variables that did not meet normality or homoscedasticity assumptions, we instead used the nonparametric Kruskal–Wallis test. Significant effects were followed by Tukey's test (for ANOVA) or Dunn's multiple-comparison test (for Kruskal–Wallis). We also fitted linear regression models with HCT as the outcome and grazed pasture in the diet (% of DMI), total DMI (kg·cow^−1^·d^−1^), and DIM as predictors. Predictors used in these models were selected based on biological plausibility and consistency across CS. Effects were expressed as minutes per unit of change, and because models were fitted to the pooled dataset, regression coefficients were interpreted as overall associations across CS rather than CS-specific effects. All statistical analyses were performed using InfoStat (Universidad Nacional de Córdoba, Córdoba, Argentina).

In S1 (spring) and S2 (summer), HCT was lower for WIN than for EXT and FC (*P* < 0.05; Figure 1a). In the same sampling windows, FC exhibited higher TS than EXT and WIN (*P* < 0.05; Table 1), a pattern consistent with advancing lactation (higher DIM in FC vs. EXT and WIN; Figure 1b; Li et al., 2019). Citrate was higher in EXT and FC than in WIN in S1, whereas pH was similar across systems (≈6.67–6.70) in S1 and S2. Thus, WIN combined the lowest citrate concentrations (Table 1) with pH values close to 6.7. pH values approaching or exceeding 6.7 place milk near the critical stability region described for type-A milks, where HCT tends to decrease as κ-casein becomes more prone to dissociate from the micelle (Anema, 2009; Huppertz, 2016). Urea content was higher in WIN in S1 (*P* < 0.05) and did not differ among CS in S2 (Table 1); although urea often improves heat stability, this effect may be offset near the minimum-pH region, which may help explain the low HCT in WIN despite higher urea (Huppertz, 2016).

**Figure 1:**
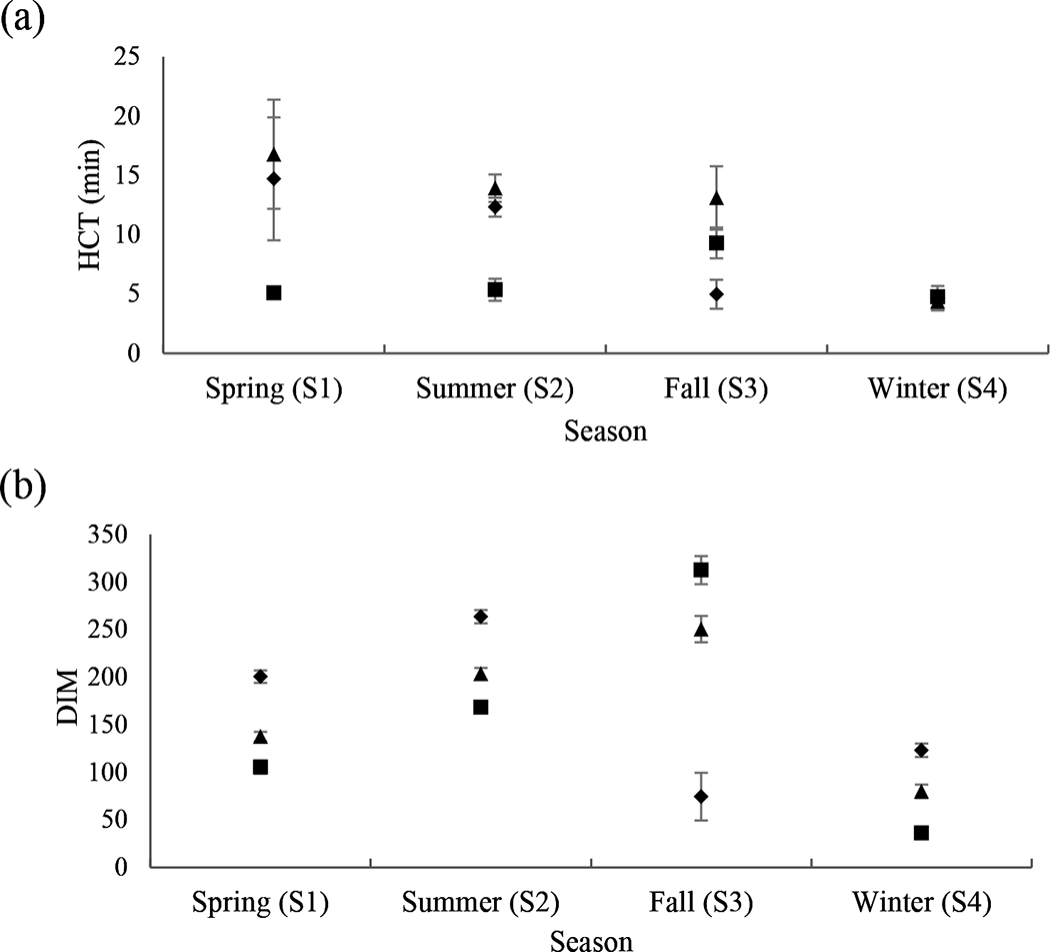
(a) Heat coagulation time (HCT) and (b) DIM of milk from cows managed under (▴) extended, (▪) winter seasonal, and (♦) fall seasonal calving systems across 4 sampling windows: spring (S1), summer (S2), fall (S3), and winter (S4). Symbols represent means and error bars indicate SD.

**Table 1:** Dry matter intake, diet composition, milk composition, and heat coagulation time (HCT) of cows managed in extended (EXT), winter seasonal (WIN), and fall seasonal (FC) calving systems across seasons^1^

Season		CS	DMI (kg/d)	PAS (% DMI)	CONC (% DMI)	FR (% DMI)	HCT (min)	TS (%)	pH	Citric acid (%)	Urea (mg/100 mL)
S1		EXT	19.5	66.7	33.3	0.0	16.8 ± 4.6^a^	13.6 ± 0.04^b^	6.68 ± 0.01^a^	0.17 ± 0.00^b^	25.7 ± 2.5^a^
		WIN	20.0	65.0	35.0	0.0	5.1 ± 0.2^b^	13.1 ± 0.20^c^	6.70 ± 0.02^a^	0.15 ± 0.01^a^	34.6 ± 0.8^b^
		FC	20.3	68.9	31.1	0.0	14.7 ± 5.1^a^	13.9 ± 0.20^a^	6.67 ± 0.02^a^	0.17 ± 0.01^ab^	26.7 ± 1.05^a^
S2		EXT	18.0	48.8	32.4	18.8	13.9 ± 1.2^b^	13.0 ± 0.04ª	6.66 ± 0.01^a^	0.16 ± 0.02^a^	30.7 ± 5.2ª
		WIN	20.1	50.1	33.3	16.6	5.4 ± 0.9^a^	13.1 ± 0.30^a^	6.69 ± 0.03^a^	0.15 ± 0.02^a^	32.8 ± 3.6^a^
		FC	16.9	24.4	35.1	40.5	12.3 ± 0.8^b^	13.5 ± 0.08^b^	6.65 ± 0.01^a^	0.14 ± 0.02^a^	29.1 ± 3.8^a^
S3		EXT	19.6	18.2	42.4	39.4	13.1 ± 2.7^c^	13.7 ± 0.80^c^	6.66 ± 0.00^a^	0.14 ± 0.02^a^	27.4 ± 6.9^b^
		WIN	18.5	26.1	33.7	40.2	9.3 ± 1.3^b^	14.9 ± 0.10^b^	6.70 ± 0.00^a^	0.16 ± 0.01^b^	29.4 ± 4.9^b^
		FC	21.1	11.0	44.8	44.2	4.9 ± 1.2ª	13.3 ± 0.05^a^	6.66 ± 0.03^a^	0.15 ± 0.02^a^	19.8 ± 1.4^a^
S4		EXT	23.1	0.0	34.8	65.2	4.4 ± 0.7ª	13.2 ± 0.2^ab^	6.72 ± 0.02^a^	0.15 ± 0.00^a^	25.7 ± 1.5^a^
		WIN	23.7	0.0	38.0	62.0	4.8 ± 0.4ª	12.9 ± 0.10^a^	6.71 ± 0.03^a^	0.15 ± 0.02^a^	26.1 ± 1.8^a^
		FC	22.0	0.0	30.9	69.1	4.6 ± 1.0^a^	13.3 ± 0.10^b^	6.73 ± 0.03^a^	0.16 ± 0.02^a^	26.3 ± 1.6^a^

a–c Within a season, values within a row with different superscripts differ among calving systems (*P* < 0.05).

1 Values are means ± SD. Pasture (PAS), concentrate (CONC), and forage reserve (FR) as percentage of total DMI; HCT = heat coagulation time at native pH.

In S3 (fall), all systems differed (*P* < 0.05), with HCT ranking EXT > WIN > FC (13.1 ± 2.7, 9.3 ± 1.3, and 4.9 ± 1.2 min, respectively; Figure 1a). The FC group had the lowest DIM and lower urea than EXT and WIN (*P* < 0.05; Table 1), consistent with reduced pasture inclusion; both features may contribute to its low stability. Notably, WIN pH was ∼6.70—often unfavorable for stability—yet citrate was higher than in EXT and FC, which could partially offset the pH effect through Ca^2+^ chelation, yielding an intermediate HCT (Anema, 2008; Huppertz, 2016).

In S4 (winter), all systems showed HCT <5 min (EXT 4.4 ± 0.7; WIN 4.8 ± 0.4; FC 4.6 ± 1.0 min) with no difference between systems for urea, citrate, or pH (*P* > 0.05; Figure 1a). All groups had pH >6.7 (Table 1), a region associated with increased κ-casein dissociation and reduced micellar stability; maximal stability is typically observed near pH 6.6–6.7 (Singh and Fox, 1986; Huppertz, 2016), and our data also showed maximal HCT in this range (Figure 2). Across windows, no single, stable predictor of HCT emerged among the measured variables: effects were context-dependent (CS × S) and influenced by DIM, milk composition, and dietary management.

**Figure 2:**
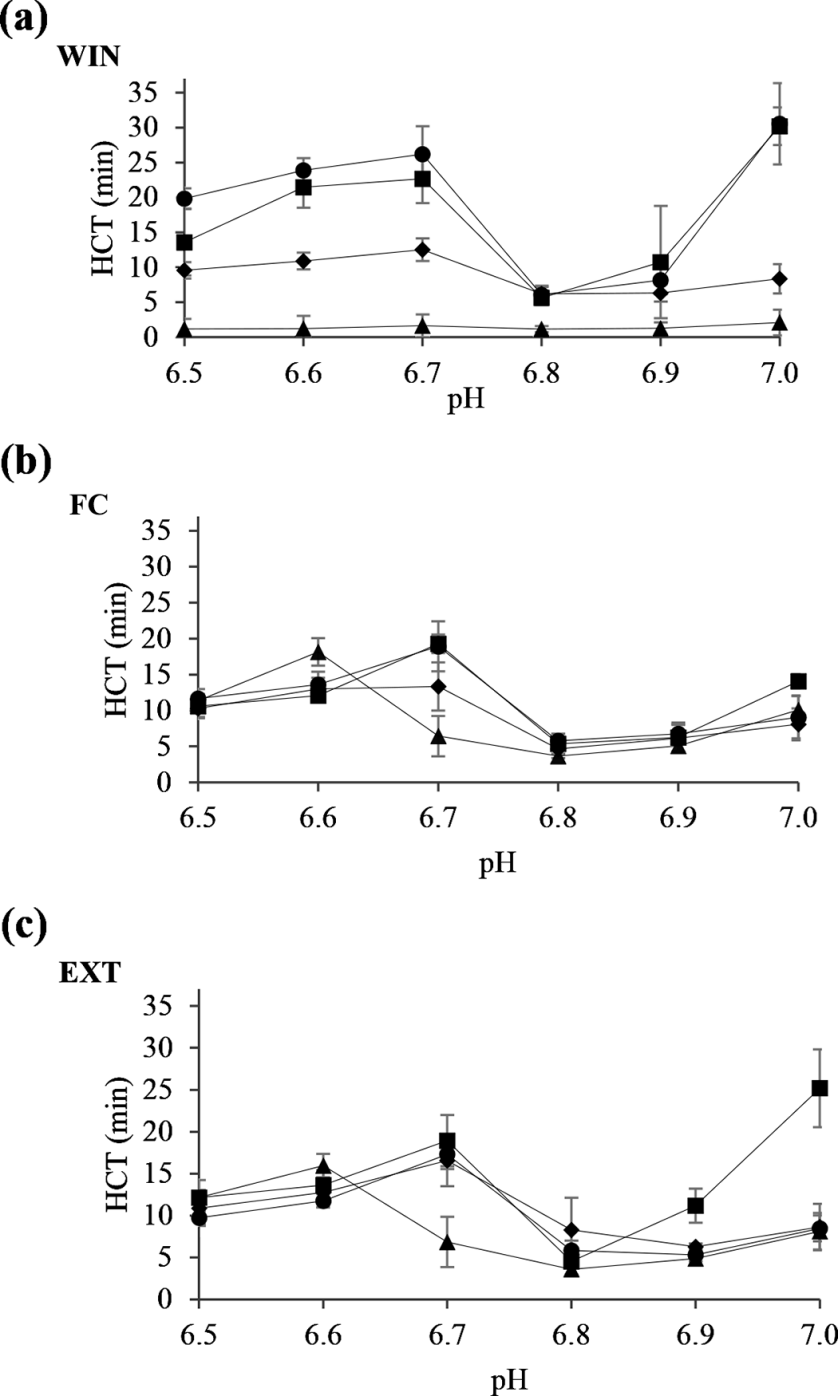
Heat coagulation time (HCT) as a function of pH within each calving system. (a) Winter seasonal (WIN), (b) fall seasonal (FC), and (c) extended (EXT) systems. Within each panel, lines represent the 4 sampling windows: spring (S1; •), summer (S2; ▪), fall (S3; ♦), and winter (S4; ▴), with symbols showing means and error bars indicating SD.

Figure 2A–2C shows the behavior of HCT as a function of pH from 6.5 to 7.0. In all combinations of CS (WIN, FC, EXT, respectively) and sampling window (S1–spring, S2–summer, S3–fall, and S4–winter), milk displayed a type-A HCT–pH profile, with maximum stability at pH 6.6–6.7 and lower stability at more acidic or more alkaline pH values (minimum around pH 6.8–6.9). Overall, HCT-pH profiles were broadly similar across CS and sampling windows (Figure 2). An exception was the winter sampling window (S4), in which the profiles shifted (Figure 2a–c): in EXT and FC, the pH associate with the peak HCT (maximum heat stability) shifted away from 6.7 relative to other sampling windows (Figure 2b,c), whereas in WIN, HCT remained <2 min across the entire pH range evaluated (Figure 2a). This pattern is consistent with the native-pH HCT results, where all systems showed HCT <5 min in S4 (winter). Notably, in S4 cows were in early lactation (DIM <120 d) and received diets with no pasture inclusion (Table 1), because winter conditions in this region severely limit the availability of grazed forage. Previous studies in pasture-based herds have associated these conditions with reduced HCT of milk (Holt et al., 1978; Kelly et al., 1982; Crowley et al., 2014; Visentin et al., 2017; Timlin et al., 2023).

Across all observations, HCT showed a positive linear association with grazed pasture in the diet (R^2^ = 0.19, *P* = 0.0093; slope = 0.07 min per percentage point) and a negative association with DMI (kg·cow^−1^·d^−1^; R^2^ = 0.32, *P* = 0.0004; slope = −1.28 min per kg). The HCT also increased with DIM (R^2^ = 0.35, *P* = 0.0002; slope = 0.03 min per day).

The overall pattern is consistent with the notion that greater access to grazed pasture is associated with higher heat stability (Holt et al., 1978). Holt et al. (1978) and Kelly et al. (1982) reported that milk produced during the grazing period (May–September) showed, on average, higher HCT than milk produced during winter, and attributed these differences to milk urea content. Similarly, in seasonal CS, Visentin et al. (2017) reported that seasonal variation in forage supply alters access to different feed sources and drives changes in milk composition relevant to HCT (e.g., urea); they also observed lower HCT at the beginning and end of lactation, which they linked to diets with reduced grazed-pasture inclusion. In contrast, Timlin et al. (2023) reported that, in synchronized CS, lactation stage (early, mid, and late lactation) is a key determinant of changes in milk functionality; accordingly, they observed increasing HCT across lactation stages, with values ranging from 8.5 to 12.6 min in early lactation, 13.7 to 16.4 min in mid lactation, and 17.8 to 20.0 min in late lactation, and they did not identify a clear relationship with nonprotein nitrogen (which includes urea). Delucchi (2010) found that HCT improves as lactation advances in Holstein cows and did not observe a late-lactation drop, attributing the pattern to increases in urea from calving to ∼180 DIM. In our dataset, no consistent urea–DIM correlation was observed.

In S4 (winter), cows were in early lactation (DIM <120 d) and had no access to grazed pasture; consistent with the simple associations observed in this study, all CS showed HCT values below 5 min (Figure 1a). However, within S4, this outcome could not be attributed to a single driver because urea, citrate, and pH did not differ among systems (*P* > 0.05), and pH values >6.7 were observed across all groups. These findings are relevant for the Uruguayan dairy industry, where milk thermal stability is important both for an export-oriented sector in which whole and skim milk powders represent a major share of dairy exports, and for domestic processing, particularly UHT milk production. According to national statistics, UHT milk production increased from 84.1 million liters in 2023 to 106.7 million liters in 2024, a 26.9% year-on-year increase (INE, 2025; INALE, 2026). More broadly, because pasture-based dairy systems with seasonally variable supplementation are common in Uruguay and in other regions with similar production schemes (e.g., New Zealand, Argentina, southern Brazil, and Paraguay), these results provide evidence of regional relevance. In practical terms, the results suggest that calving strategy alone is unlikely to be a sufficiently reliable lever for managing milk heat stability and those processors should be particularly alert to a greater risk of low HCT during winter-type conditions characterized by early lactation and reduced pasture access. Overall, the study helps frame HCT as a multifactorial trait in pasture-based systems and supports the need for future controlled cow-level studies, including differential feeding trials and more detailed physicochemical measurements (e.g., ionic Ca and available citrate), to identify actionable determinants of milk thermal stability
